# Thank you to our authors, reviewers, and readers

**DOI:** 10.1038/s42003-018-0138-z

**Published:** 2018-09-06

## Abstract

A year has passed since *Communications Biology* opened for submissions. We’d like to take this opportunity to look back on the past year and thank all those who have contributed to the journal.

For the editorial staff of *Communications Biology*, the year since we opened for submissions has passed in a blur. We’ve received more than 850 submissions and, although many of our papers are still making their way through the peer review process, we have already published well over 100 papers. Meanwhile, we are busy working toward the goals set out in our launch editorial^1^: to be an inclusive, multi-disciplinary journal for all biologists and to provide the best author service we possibly can.

We are happy to say that we are on the right track. Our published papers represent the breadth of the life sciences—from biophysics to entomology, cognitive neuroscience to microbiology, and much more. Our in-house editors and Editorial Board Members have worked hard to meet our goals of delivering timely decisions and maintaining communication with our authors throughout the editorial and review process. And where we have fallen short of our goals, we strive to do better.

But we wouldn’t be anywhere today without our authors, reviewers, and readers. We have had the privilege to publish truly outstanding, high-quality papers that, judging by the response of our reviewers and readers, are important contributions to their respective fields of research. We want to take this moment to thank all of our authors for entrusting us with their discoveries.

We are grateful to the more than 1100 individual reviewers who have contributed to the success of our journal and the quality of the final published papers you read in our pages. Our reviewers, like our authors, come from all over the world—representing more than 40 countries—and all career stages^2^. We believe that to truly have an inclusive journal, our reviewers and editors should reflect the broader biological community, and we keep this guiding principle in mind in everything that we do. Our editors consider all forms of diversity when inviting potential reviewers and routinely ask invited reviewers to consider gender balance, ethnic and geographical diversity, and career stage diversity when suggesting alternative reviewers in the event that they must decline our invitation. Despite these efforts, we find that only 26% of our reviewers are women; we are actively working toward increasing this proportion. As part of this effort, we call on our authors to consider diversity when suggesting reviewers for their submitted papers.

We are grateful to the more than 1100 individual reviewers who have contributed to the success of our journal and the quality of the final published papers you read in our pages.

With Peer Review Week just around the corner—the theme of which we are happy to see is ‘Diversity and Inclusion in Peer Review’—we would also like to take this opportunity to acknowledge our reviewers more publicly. Starting this month, we will highlight a Reviewer of the Month to recognize some of our most outstanding reviewers. Each month, one of our editors will be tapped to choose a reviewer they feel has made a significant and positive contribution to the peer review process, regardless of whether the paper was eventually accepted by the journal. We are not choosing our Reviewers of the Month based on speed, as we feel that an outstanding review is worth the wait (within reason of course). Rather, we are looking for those reviewers who take both a broad and detailed view of the paper, who demonstrate professionalism and compassion, and provide comments that truly help the authors to improve their work. We embark on this project with no small amount of trepidation. After all, many of our reviewers are outstanding and all of them are important to the success of our authors and the journal. But we hope that by publicly recognizing even a small number of our reviewers, we can help humanize the often impersonal process of peer review.

Finally, in honor of the upcoming Peer Review Week and outstanding reviewers everywhere, we’d like to hear your stories of peer review done right. Tell us about a time that a review helped you to improve the paper, or when a comment from a reviewer simply brightened your day. Use the comments below or email us at commsbio@nature.com and we may share your story in a future editorial.
